# Chemical composition and antioxidant activity of some Syrian wild mushroom (*Agaricus* spp) strains

**DOI:** 10.1038/s41598-023-43265-w

**Published:** 2023-09-23

**Authors:** Boushra Hola, Ramzi Murshed, Mouwafak Jbour

**Affiliations:** 1 Department of Horticulture, Faculty of Agriculture, Damascus University, Damascus, Syria; 2 General Commission for Scientific Agricultural Research (GCSAR), Al Halboni, Libraries Street, Damascus, Syria

**Keywords:** Microbiology, Fungi, Fungal ecology

## Abstract

This research aims to study the chemical content (moisture, ash, fat, protein, fiber and carbohydrate), phenolic compounds, and antioxidant activity of the fruit bodies resulting from the cultivation of six edible Syrian wild mushroom strains of the *Agaricus* genus. These strains were collected from the western countryside of Homs governorate in Syria (*Agaricus bispours* BR5, *Agaricus bispours* B.R.9, *Agaricus sinodeliciosus* BR17, *Agaricus qilianensis* BR22, *Agaricus sinodeliciosus* BR42 and *Agaricus qilianensis* BR47) and were compared to the commercially cultivated *Agaricus bisporus* strain Sylvan A15 as a control. The results showed that wild strains had a good chemical composition. The BR47 had the highest protein content among the studied strains (29.52%), which was close to the content of the control (28.55%). All strains recorded higher carbohydrate content compared to the control (p < 0.01), and BR42 had the highest content (72.24%). The fat content in the studied strains ranged from 1.68 to 5.34%, and they were all less than the control (7.29%). BR9 was marked by a high phenol content (1.93 mg.g^–1^ of dry weight), while the control had higher antioxidant activity (82.41%). A strong correlation was noted between antioxidant activity, protein, fat and ash. Some studied strains showed nutritional value and distinctive biological properties, indicating they can be used for food and pharmaceutical purposes.

## Introduction

Fungus is a non autotrophic organism that grows in all types of soils, forests, fields, mountains, hills, deserts, deadwood logs, or similar decomposted organic residues^1^. The concept that diet is fundamental to human health has led to increased consumer demand for nutritional supplements and functional foods^2,3^.

Mushroom-based nutritional supplements or functional foods have become very attractive recently^4,5^. Although mushrooms currently do not constitute a large part of a human diet, their consumption continues to increase due to their high nutritional value.

Apart from minerals, fibers, fatty acids, and essential amino acids present in them, they contain a broad range of vital compounds with nutritional and medical properties^6^, such as phenolic compounds, polyketides, terpenes, steroids^7^, beta-carotene, and vitamins A and C^8^. The mushroom is distinguished by a range of vital activities such as anti-microbial activities^9–11^, and antiviral activities^12^. Several studies consider mushrooms as an easy source for obtaining phenols, vitamins and other natural antioxidants^13,14^, and perhaps the synergy of these components with each other is the main reason of the beneficial effects described in clinical trials^15,16^.

Several species of edible wild mushroom have high levels of proteins, vitamins, and minerals, such as magnesium, calcium, sodium, iron, zinc, selenium, etc., and low calorie values^17,18^. It also contains dietary fibers, especially chitin and beta glycan responsible for these functional properties^19^. Recent scientific studies^20,21^ confirm that bioactive compounds from many species of edible mushrooms are involved in lowering blood cholesterol levels, protect against various disorders including tumor, such properties directly or indirectly associated with their high antioxidant activity.

Traditionally, mushrooms of the *Agaricus* spp. genus have been used to treat many common conditions including atherosclerosis, hepatitis, increased blood grease, diabetes, dermatitis and cancer^22^. It also contains immune, antibacterial and antitumor properties^23,24^, and has antioxidant properties and contains phenols, argotheonin and minerals^25^. However, studies that characterize the wild species of this genus in Syria were not found. The aim of this study is to assess the chemical composition, total phenols, and antioxidant activity of some edible wild species of *Agaricus* spp. found in Homs governorate, central Syria.

## Results and discussion

### Chemical composition

The results of the chemical analysis of the fresh mushroom samples showed that moisture content ranged from 88.72% in BR47 strain to 93.03% in BR42 strain (Table 1). All strains excluding BR47, had significantly higher average of moisture content compared to the control. These results were consistent with the results of Rahi and Malik^26^, who confirmed that the percentage of moisture in edible mushrooms was between 85 and 95%, as well as the results of several previous studies on *Agaricus bispaurs*^22,27^. The moisture content of mushrooms is influenced by several factors, such as species, environmental conditions, and other factors such as harvesting, preparation and storage^28,29^.

**Table 1:** Moisture and ash content in studied mushroom strains.

Strain	Moisture (% of fresh weight)			Ash (% of dry weight)		
	Cap	Stipe	Average	Cap	Stipe	Average
BR5	90.31 ± 0.30efgh	90.24 ± 0.24defgh	90.28C	5.24 ± 0.16g	5.08 ± 0.09g	5.16E
BR9	91.11 ± 0.31gh	90.65 ± 0.15fgh	90.88C	5.43 ± 0.28g	8.99 ± 0.08d	7.21D
BR17	89.35 ± 0.22bcde	89.07 ± 0.34bcd	89.21B	9.94 ± 0.16c	9.82 ± 0.34cd	9.88C
BR22	91.39 ± 0.37h	90.13 ± 0.63cdefg	90.76C	7.74 ± 0.18e	6.41 ± 0.54f	7.08D
BR42	92.89 ± 0.15i	93.16 ± 0.17i	93.03D	5.74 ± 0.03fg	5.21 ± 0.11g	5.48E
BR47	88.97 ± 0.30bc	88.47 ± 0.42b	88.72AB	9.70 ± 0.18cd	11.34 ± 0.22b	10.52B
Control	89.68 ± 0.52cdef	86.83 ± 0.17a	88.25A	10.28 ± 0.10c	13.51 ± 0.18a	11.90A
Average	90.53B	89.79A		7.72B	8.62A	
L.S.D0.01%	Interaction: 1.21	Part: 0.46	Strain: 0.85	Interaction: 0.88	Part: 0.33	Strains: 0.62
CV	1.1			5.6		

Small letters indicate that there are significant differences between the average values of studied parts by strain.

Capital letters indicate that there are significant differences between the average values of the studied strains or between the averages of the studied parts.

Ash is an important chemical parameter and reflects the nutritional mineral content. Essential mineral elements play a vital role in the proper development of human health, and Amin et al*.*^1^, found that the wild *A. bisporus* fungus content of ash reached 53 g per 100 g of dry weight. Table 1 showed that ash content in the mushroom samples ranged from 5.16 to 11.90%, with significant differences between the control and the wild strains (p < 0.01). The ash content of the control was significantly higher than that of the studied wild strains. Oboh and shodehiden^30^ reported that ash content of three edible wild mushroom species in Nigeria ranged from 31.7 to 17.5% of dry weight in the cap and from 19.6 to 12.3% of dry weight in the stipe.

The average ash content of stipe was significantly higher (8.62%) than it of cap (7.72%) (Table 1). BR17 and BR47 had caps with good ash content without significant differences compared to that of the control. These results are in line with the results of Nasiri et al.^31^, who noted significant differences in ash content between the cap and stipe of *A. bisporus* mushroom. According to Oluwafemi et al.^32^ the ash content of the capwas higher than it in the stipe of *Plueotus ostreatus* mushroom. This difference may be attributed to the differences between the species that were studied, the media of growth, and the environmental conditions.

The average fat content in studied strains ranged from 1.62 to 5.34% of dry weight, with significant differences among them and the control (Table 2). The fat content in the control was higher than it in the wild strains (7.29%). Obtained results were higher than the results of Atila et al.^33^, who found that the content of fat in *Agaricus bisporus* was 1.56% of dry weight. Barros et al.^34^ found that the fat content in *Agaricus arvensis* was 0.14% of dry weight, and Barros et al.^35^ reported that the fat content in *A. bisporus* mushrooms was 0.92% of dry weight without significant differences between cap and stipe. The results showed that fat content of both cap and stipe in the studied strains was 4.16% and 4.18%, respectively, which is higher than the results of Nasiri et al.^31^, who found that the content in both cap and stipe of *Agaricus bisporus* mushroom was 2.48 and 2%, respectively. Studied strains varied in terms of which part, cap or stipe, has higher content of fat. BR9 and BR47 were in line with the findings of Oboh and shodehiden^30^ that cap had higher fat content than stipe in three species (*Termitomyces robustus*, *Coprinus* sp, and *Volvariella esculenta*). However, in BR17 and BR22, the stipe had a higher fat content than the cap. Despite the fact that mushrooms have a low-fat content, they contain unsaturated beneficial fatty acids^36^.

**Table 2:** Fat and protein content (% of dry weight) in studied strains.

Strains	Fat (% of dry weight)			Protein (% of dry weight)		
	Cap	Stipe	Average	Cap	Stipe	Average
BR5	5.58 ± 0.30de	5.09 ± 0.35e	5.34B	21.01 ± 0.133ef	19.95 ± 0.095f	20.48B
BR9	5.70 ± 0.42cde	0.72 ± 0.20g	3.21D	24.32 ± 0.109d	19.30 ± 0.313f	21.81B
BR17	1.22 ± 0.15g	5.92 ± 0.11cde	3.57D	28.93 ± 0.667ab	27.65 ± 0.203bc	28.29A
BR22	1.24 ± 0.17fg	6.28 ± 0.27bcd	3.76CD	20.12 ± 0.747f	23.05 ± 0.518de	21.58B
BR42	1.27 ± 0.12fg	2.10 ± 0.06f	1.68E	14.52 ± 0.139g	11.54 ± 0.219h	13.03C
BR47	6.53 ± 0.09bc	2.11 ± 0.18f	4.32C	28.63 ± 2.542b	30.41 ± 0.587ab	29.52A
Control	7.56 ± 0.11a	7.01 ± 0.23ab	7.29A	25.30 ± 0.088cd	31.80 ± 0.532a	28.55A
Average	4.16A	4.18A		23.26A	23.39A	
L.S.D 0.01%	Interaction: 0.87	Part:0.33	Strains: 0.62	Interaction: 2.91	Part: 1.10	Strains:2.06
CV%	10.9			6.5		

Small letters indicate that there are significant differences between the average values of studied parts by strain.

Capital letters indicate that there are significant differences between the average values of the studied strains or between the averages of the studied parts.

Mushrooms are good sources of high quality proteins^37^. Table 2 show that the content of protein in the studied strains ranged from 13.03 to 29.52% of dry weight. BR47 and BR17 recorded the highest content (29.52 and 28.29%, respectively), without significant differences compared to the control (28.55%). Previous studies reported that the raw protein content of *Agaricus bisporus* and *Agaricus bitorquis* were 16.4 and 19.53%, respectively^38,39^, which are less than most of the values reported in this study. In comparison, Tsai et al.^27^ found that the content of protein in *Agaricus bisporus* ranged from 21.3 to 27% of dry weight. There were no significant differences between cap and stipe contents, however, this was strains dependent. Cap had significantly higher protein content than stipe in case of BR9 and BR42 , which is consistent with the results of (Oboh and shodehiden; Nasiri et al.)^30,31^ who stated that the cap’s protein content in the *Agaricus bisporus* mushroom is much higher than in the stipe. BR22 was distinguished by its higher content in the stipe. The content of protein in edible wild mushrooms is influenced by several factors such as species, stage of growth, part of the sampling, and location^34^.

Mushrooms are an important source of beneficial dietary fiber for health^40^. The studied strains had varied content of fiber. BR17 content (16.06%) was insignificantly higher than it of the control (15.66%). The second highest fiber content was found in control and BR47 (Table 3). Amin et al.^1^found that the content of fiber in *A. bisporus* was 17.76% of dry weight, and Tsai et al.^27^ stated that the fiber content in the mushroom *Agaricus bisporus* ranged from 23.3 to 17.7%, which is higher than the results of this study. Also, Mohiuddin et al.^41^ found that the dietary fiber content in mushrooms ranged from 71.51% for *Agaricus bisporus* to 63.44% for *Pleurotus ostreatus*. Mushroom content of fiber depends on the species, maturity of the fruiting bodies and substrate^42^.The results demonstrated significant differences in the content of fiber between the cap and the stipe, where the higher content of fiber was in the stipe. This is due to the higher cellulose content in the stipe as compared to the cap^32^. These results are consistent with the results of Oboh and shodehiden^30^. Nasiri et al.^31^ studied the fiber content of the cap and stipe of the *Agaricus bisporus*, and it was 31.11 and 38.08%, respectively. These are higher than the value obtained in this study.

**Table 3:** Fiber and total carbohydrates content (%) in the studied mushroom strains.

Strain	Fiber (% of dry weight)			Carbohydrates (% of dry weight)		
	Cap	Stipe	Average	Cap	Stipe	Average
BR5	11.75 ± 0.364ef	13.81 ± 0.579d	12.78C	56.42 ± 0.324c	56.07 ± 0.930c	56.24B
BR9	11.21 ± 0.435fg	14.19 ± 0.176d	12.70C	53.35 ± 0.759c	56.79 ± 0.361c	55.07B
BR17	13.50 ± 0.579d	18.61 ± 0.297b	16.06A	46.41 ± 0.733d	38 ± 0.256f	42.21C
BR22	9.91 ± 0.246gh	16.55 ± 0.215c	13.23C	60.99 ± 0.324b	47.71 ± 0.930d	54.35B
BR42	6.93 ± 0.174j	8.21 ± 0.070ij	7.57D	71.54 ± 0.255a	72.95 ± 0.198a	72.24A
BR47	13.10 ± 0.300de	16.70 ± 0.511c	14.90B	42.05 ± 2.669e	39.45 ± 0.778ef	40.75C
Control	9.07 ± 0.267hi	22.25 ± 0.659a	15.66AB	47.79 ± 0.200d	25.43 ± 1.224g	36.61D
Average	10.78B	15.76A		54.08A	48.06B	
L.S.D 0.01%	Interaction:1.43	Part:0.54	Strain: 1.01	Interaction: 3.45	Part: 1.30	Strain: 2.44
CV %	5.6			3.5		

Small letters indicate that there are significant differences between the average values of studied parts by strain.

Capital letters indicate that there are significant differences between the average values of the studied strains or between the averages of the studied parts.

Carbohydrates is the largest nutritional component of mushrooms^40^. The results showed that all the wild strains had higher carbohydrate content than that of the control (36.61% of dry weight). BR42 had the highest content (72.24%) while the lowest content was in BR22 (54.35%) (Table 3). Previous results showed that the content of carbohydrates in mushrooms ranged from 35 to 75% of dry weight, and mostly in the formof polysaccharides like chitin, β-glucans, and trehalose^43^. The content of carbohydrates in mushrooms varied among species. It was 56.47% in *Agaricus bisporus* compared to 39.94% in *Agaricus bitorquis*^38^. Barros et al.^35^ stated that *Agaricus bisporus* had a content of 8.25 percent. Other studies^44^ reported a wide range (13 to 65 percent of the dry weight) of carbohydrates content in *Agaricus* spp. species, however, Tsai et al.^27^ found that carbohydrates in *Agaricus bisporus* ranged from 38.3 to 48.9 percent of the dry weight depending on the stages of growth.

Significant differences between the content of carbohydrates in the cap (54.08%) and in the stipe (48.06%) were found, but the content of both the cap and the stipe varied from one strain to another. Nasiri et al.^31^ studied the carbohydrate content in both the cap and the stipe of the *Agaricus bisporus* mushroom, and found that the stipe had significantly higher (31.41%) content than the cap’s (20.59%), yet these are less than the results of this study. The current findings are inconsistent with the results of Oboh and shodehiden^30^, who found that the content of carbohydrates in the cap to be less than it in the stipe. This can be explained by the differences in growth conditions, genetic factors, geographical differences, and methods of analysis^45,46^. Mushroom content of carbohydrates is influenced by several factors, such as species, stage of growth, part of sampling, available level of nitrogen and location^47^.

### Total phenolic compounds and antioxidant activity

Phenols are one of the bioactive compounds with benefits for human health. They are found in mushroom with good concentrations^48,49^. The content of total phenols in the fruiting bodies of the studied strains (Table 4) ranged from 1.93 to 0.58 mg gallic acid equivalents (GAE).g^–1^ dry weight. This is less than the content indicated by Prasad et al.^50^, who found that total phenols in mushroom ranged from 6.08 to 24.85 mg GAE.g^–1^ dry weight. This can be explained by the fact that wild mushroom contains higher content of phenols than cultivated mushroom^51^. The results demonstrated also that the phenolic content of the BR9 strain (1.93 mg.g^–1^) was higher than it in the other studied strains, followed by control (1.42 mg.g^–1^). The lowest content registered in BR42 (0.25 mg.g^–1^). Both trans-cinnamic and chlorogenic acids are the most important phenolic acids found with high concentrations in *Agaricus* mushrooms^52^. According to Mujić et al.^53^, the content of phenolic compounds in mushrooms ranged from 7.8 to 23.07 mg GAE.g^–1^ dry weight. Alispahić et al.^48^ reported that the content of phenols in edible mushrooms ranged from 4.94 to 35.56 mg GAE.g^–1^ dry weight, and this difference can be attributed to variation in the environmental conditions surrounding the fungus at the collection site and extraction method^54^. The data in Table 4 indicated that the content of the cap and stipe was, strain-depending. Thus the content of phenols in the cap was significantly higher than it in the stipe in case of BR5, BR9 and BR47, and vice versa in the rest of the strains and the control.

**Table 4:** Total phenolic content (mg GAE.g^–1^dry weight) and antioxidant activity (%) in studied mushroom strains.

Strain	Total phenolics (mg.g^–1^ dry weight)			Antioxidant activity (%)		
	Cap	Stipe	Average	Cap	Stipe	Average
BR5	0.94 ± 0.017f	0.82 ± 0.020g	0.88F	78.33 ± 0.310c	6.20 ± 0.922i	57.26C
BR9	3.08 ± 0.023a	0.78 ± 0.018gh	1.93A	34.83 ± 0.284i	53.33 ± 0.545f	44.08E
BR17	0.99 ± 0.015f	1.48 ± 0.030d	1.23C	63.69 ± 0.850d	82.12 ± 0.454ab	72.91B
BR22	0.41 ± 0.013i	0.75 ± 0.017h	0.58F	60.59 ± 0.341de	50.90 ± 1.367fg	55.75C
BR42	0.09 ± 0.008j	0.40 ± 0.011i	0.25G	45.91 ± 0.844h	48.76 ± 0.580gh	47.34D
BR47	1.91 ± 0.008b	0.41 ± 0.008i	1.16D	84.25 ± 0.489a	58.59 ± 0.386e	71.42B
Control	1.15 ± 0.009e	1.69 ± 0.023c	1.42B	84.09 ± 1.605a	80. 74 ± 0.520bc	82.41A
Average	1.22A	0.90B		64.53A	58.66B	
L.S.D 0.01%	Interaction: 0.06	Part: 0.02	Strains: 0.04	Interaction: 3.18	Part:1.20	Strains: 3.18
CV%	3			2.3		

Small letters indicate that there are significant differences between the average values of studied parts by strain.

Capital letters indicate that there are significant differences between the average values of the studied strains or between the averages of the studied parts.

The DPPH (2,2-diphenyl-1-picrylhydrazyl) is a stable free radical that can be used to measure the radical scavenging activity of antioxidant of specific compounds or extract in a short time. The studied strains showed good scavenging activity of DPPH and the antioxidant activity ranged from 44.08 to 82.41% (Table 4). The control had the highest activity, followed by two wild strains of BR17 and BR47. Atila et al.^33^ and Khan et al.^55^ indicated that *A. bisporus* mushroom had a higher antioxidant activity than other species (*P. eryngii*, *Grifola frondose*, *P. ostreatus*, and *L. edodes*). The results showed higher antioxidant activity in the cap compared to it in the stipe, and this varied from one strain to another. Such activity can be explained by the presence of some organic acids, such as citric acid, malic acid and quinic acid with good and higher concentrations in the cap than that in the stipe^56^.

#### Correlations between chemical parameters

There were a strong positive correlations between the content of protein and fiber (r = 0.959), ash and protein (r = 0.888), and ash and fiber (r = 0.780) (Table 5). The antioxidant activity correlated positively with protein (r = 0.833) and fat (r = 0.721), which could be attributed to the presence of unsaturated fatty acids such as linoleic, linolenic and Sulphur amino acids such as ergothioneine, that has antioxidant potential^57^. A strong correlation between antioxidant activity and ash was found (r = 0.872) (Table 5). This is might be explained by the high amounts of mineral elements in mushroom, of which zinc, selenium and copper are the most important; these minerals are involved in the synthesis of antioxidant enzymes and thus protect living cells from the effect of free radicals^58^. Carbohydrates negatively correlated with fat, protein, fiber, ash and antioxidants activity.

**Table 5:** Correlations among the different compounds of studied mushroom strains.

	Fat	Protein	Fiber	Ash	Carbohydrates	Phenolate	Antioxidant activity
Fat	1						
Protein	0.616	1					
Fiber	0.664	0.959	1				
Ash	0.560	0.888	0.780	1			
Carbohydrates	− 0.718	− 0.987	− 0.960	− 0.906	1		
Phenolate	0.373	0.597	0.602	0.495	− 0.589	1	
Antioxidant activity	0.721	0.833	0.779	0.872	− 0.876	0.218	1

## Conclusion

The chemical composition of the studied wild strains revealed remarkable variations between them, and among them and the control. BR17 and BR47 had a good content of protein, fiber and ash compared to the control, and these chemical components were positively associated with antioxidant activity. The studied strains varied in terms of the content of the various chemical compounds found in the cap and stipe. It was clear that the stipe had a high nutritional value that could not be ignored or wasted, therefore, it should be used as an important food source, either in a dried form that would enter into the preparation of soups or other food products, or in its fresh form.

## Materials and methods

### Collection and cultivation of mushroom strains

The research was conducted in the labs of the General Commission for Scientific Agricultural Research during 2021 and 2022. The chemical composition of fruiting bodies, which resulted from cultivation of six wild local strains of mushroom (BR5, BR9, BR17, BR22, BR42 and BR47), was studied. These wild strains were collected from various forests and grasslands in the western countryside of Homs governorate, Syria (latitude: 34° 42′ 18" to 34° 51′ 34.0″ North, longitude: 36° 21′ 40.4" to 36° 33′ 04.5" East, altitude 533 to 757 m above sea level), and described both morphologically and molecularly and submitted to the Genbank (Table 6). The commercially cultivated *Agaricus bisporus* strain Sylvan A15 was used as a control.

**Table 6:** The Genbank taxonomy and accession numbers of collected wild strains.

Collection no	Accession numbers	Taxonomy
B.R.5	*OP648153*.1	*Agaricus bisporus*
B.R.9	OP648154.1	*Agaricus bisporus*
B.R.17	OP648155.1	*Agaricus sinodeliciosus*
B.R.22	OP648156.1	*Agaricus qilianensis*
B.R.42	OP648157.1	*Agaricus sinodeliciosus*
B.R.47	OP648159.1	*Agaricus qilianensis*

The species were cultivated on traditional compost according to Sithole et al*.*^59^. Commercial production of *A. bisporus* is carried out by cultivation on a composted mixture based on wheat straw, horse or chicken manure, gypsum, and water^60^. The composting process involves two phases (I and II). In phase I, the straw is first wettened with water and subsequently mixed with the other components. This phase lasts 15–21 day^60^, during which the compost temperature increases to 80 °C due to thermophilic microorganisms. Subsequently, a pasteurization process (phase II) is performed. The compost is conditioned at 45–50 °C for about 4–9 days until the ammonia level becomes non-toxic to *A. bisporus* mycelia, after which the temperature is reduced to about 25 °C^60^. At the end of this stage, compost can be used for (optimal) *A. bisporus* growth^59^. The *A. bisporus* mushroom grows under a controlled environment with a regular room temperature of 22.5 ± 0.5 °C and compost temperature of 27–24 °C^59^. After maturity (4–5 weeks of cultivation) the fruit bodies were picked and transported to the laboratory for analysis^59^.

### Preparation of samples

The mushrooms were cleaned, then the cap was separated from the stipe, cut into small pieces (0.5 cm^2^), and dried in an oven (J P Selecta S.a. Spain) at 55 °C to constant weight. The samples were kept in a dry place until they were analyzed.

### Chemical analyses

The AOAC (Association of Official Analytical Chemists) methods No. 925.10, 942.05, 950.36, 96,315, and 973.18 were used to determine the content of moisture, ash, protein, fat, carbohydrates, and fiber of the mushroom samples^61^. Five marketable mushroom fruits from the first flush of each of the four replications (plastic bags of 0.1 m^2^) were collected at the same growth phase (just before the veil opening) to prepare the needed quantities for these analyses.

Moisture content was estimated by drying the samples in the previous oven dryer (J P Selecta S.a. Spain) at 105 °C until a constant weight is reached. The ash content was determined by heating the sample in a furnace (HOBERSAL, SPAIN) at 550 °C for 3 h. The total nitrogen in samples was determined using the Kledahl method (GERHARDT, Germany), and the protein content was estimated based on the nitrogen content (N × 6.25)^6^. The fat content was estimated using the Soxhlet method (Vissal, India) and using the hexane as a solvent for extraction. The crude fiber was evaluated by digestion of the samples by washing them with acid (H_2_SO_4_), then with alkaline (KOH), after that drying the samples at 105 °C for 6 h, then incinerating them at 600 °C.

The content of the carbohydrate was determined by the following equation^40^:

$$\mathrm{\%}\,\,carbohydrate=100-\left(Moisture+fat+protein+fiber+ash\right).$$

### Estimation of total phenolic compounds and antioxidant activity

#### Preparation of methanolic extract

The methanolic extract was prepared according to the method of Keleş et al.^62^ with some modifications. One gram of mushroom`s dried powder was mixed with 10 ml methanol (80%) and stirred for 1 h at room temperature. The mixture was filtered using filtration paper (MACHEREY–NAGEL No.1 paper) and stored at − 18 °C until use.

#### Determination of total phenolic compounds

The methanolic extract (1 mL) was mixed with Folin-Ciocalteu reagent (500 μl) diluted in water (1:10); after 3 min 1 ml of sodium carbonate (10%) was added to the mixture and completed to 10 ml with distilled water. The final solution was left in the dark for 1 h at room temperature, the absorbance was measured using the Spectrophotometer (T80 + UV/VIS, Britain) at 765 nm and the gallic acid was used to prepare a standard curve with a concentration ranging from 25 to 75 mg.l^–1^. The result was expressed as mg gallic acid equivalents (GAE).100 g^–1^ dry weight^63^.

#### Determination of antioxidant activity

The antioxidant activity was evaluated using the DPPH (2,2-diphenyl-1-picrylhydrazyl) method according to the procedure reported by Jacinto-Azevedo et al.^64^, with some modifications. One ml of methanolic extract was mixed with 3.9 ml of methanolic DPPH solution (0.2 mg.100 ml^–1^). After 30 min in the dark, the absorbance was measured at 517 nm, and the % inhibition was calculated based on the following equation

$$\mathrm{\% }\,\,Inhibion= \frac{\mathrm{ Absorbance\,\, of \,\,the\,\, control}-\mathrm{Absorbance \,\,of\,\, the\,\, }sample}{\mathrm{ Absorbance\,\, of\,\, the\,\, control }}\times 100.$$

### Statistical analysis

All analyses were performed in four replicates. The data were expressed as means and analyzed by using Gen Stat.12 statistical program. A factorial design and analysis of variance (ANOVA) were used in the experiment, followed by Fisher Least Significant Difference (LSD) test to evaluate the significant difference between means (P < 0.01).

## Data Availability

The datasets generated during the current study are available on the online repository [http://www.ncbi.nlm.nih.gov/taxonomy], accession numbers areas the following:

**Table:** 

Collection no.	Accession numbers
B.R.5	OP648153.1
B.R.9	OP648154.1
B.R.17	OP648155.1
B.R.22	OP648156.1
B.R.42	OP648157.1
B.R.47	OP648159.1
